# Assessment of Cancer Patients' Relatives' Knowledge, Perception, and Attitude Toward Cancer

**DOI:** 10.7759/cureus.43457

**Published:** 2023-08-14

**Authors:** Mihika Deshpande, Mrunal Meshram, Priyanka Paul, Amit Reche, Rahul R Bhowate, Anvika Deshpande, Shreyash Borkar

**Affiliations:** 1 Department of Public Health Dentistry, Sharad Pawar Dental College and Hospital, Datta Meghe Institute of Higher Education and Research, Wardha, IND; 2 Department of Oral Medicine and Radiology, Sharad Pawar Dental College and Hospital, Datta Meghe Institute of Higher Education and Research, Wardha, IND

**Keywords:** attitude, perception, knowledge, decision-making, treatment, tobacco, oral cancer

## Abstract

Background

Assessment of cancer patients' relatives' knowledge, perception, and attitude regarding cancer. To spread awareness about oral cancer in areas with a high prevalence of cancer, aid in its prevention, and accelerate the treatment to facilitate early-stage disease elimination and improve prognosis.

Material and methods

A cross-sectional study conducted at a hospital was done, where 23 questions were posed to the relatives of the patients admitted to the hospital. The questions were formulated to assess the knowledge of relatives regarding cancer, their perception, and their attitude toward the decision-making and treatment protocols. The questions also included information about the patient's habits and associated problems with it. A total of 400 relatives participated in the study, where all participants were adequately informed beforehand, and their written consent was taken before proceeding with the questions. All the questions were formulated in the native language that they could easily understand.

Result

The study found that participants had limited knowledge about the causes, treatment, prevention, and recurrence of oral cancer. Additionally, the family members were unsupportive of therapy and delayed seeking medical care.

Conclusion

It is crucial to raise awareness about oral cancer and inform individuals about available treatments, government programs, and counseling services to aid them in decision-making.

## Introduction

According to the American Cancer Society (ACS), a group of diseases is characterized by uncontrolled growth and spread of abnormal cells. The term "cancer" came from Ancient Greek, meaning "crab" because, like crab cancer, cells seem to "grab on and won't let go" [1]. It will account for around 10 million fatalities in 2020, or roughly one in every six, making it the world's biggest cause of mortality. Smoking, having a high body mass index, drinking, eating fewer fruits and vegetables, and not exercising cause around one-third of cancer deaths. The estimated mortality due to cancer in India was 770,230 in 2020, and it increased to 789,202 in 2021 and to 808,558 in 2022. India's cancer burden is projected to grow to over 15 lakh cases by 2025 [2].

Oral cancer is the sixth most prevalent type of cancer worldwide. Oral cancer is an even greater issue in India than it is in the West [3]. Modernization and lifestyle changes have worsened the eating habits of people, which led to adopting unhealthy living habits, which has majorly contributed to the increase in cases of oral cancer. The population increase and aging of low- and middle-income countries, as well as the westernization of their lifestyles, are currently their biggest issues. These factors make the objective of cancer prevention programs in these nations crucial. However, a high level of understanding of the mechanism of cancer and awareness of early symptoms is required to build effective preventive programs. Additionally, there ought to be favorable attitudes toward screening initiatives. Culture, myths, and taboos are just a few of the variables that can impact early detection and appropriate care. Even with sufficient resources, the success of any cancer treatment program might be hampered by a failure to recognize these internal barriers [1,2]. In India, friends and family are crucial to the patient's care as well as the treatment's decision-making process [4]. It has been found that relatives play a major role in every step of the treatment, from deciding on the doctor and the hospital to deciding on the surgical procedure. It was also found that the relatives even decide whether to tell the full truth to the patient about their health condition or not as it will affect the mental health of the patient. Studies have previously been published in the literature on how family engagement in decision-making results in higher satisfaction among patients and regular treatment follow-ups [5-7].

This cross-sectional analysis was therefore carried out to evaluate the knowledge, perception, and attitudes of healthy relatives of cancer patients who came to visit their ill relatives at Siddharth Gupta Memorial Cancer Hospital, Datta Meghe Institute of Higher Education and Research (DMIHER), Sawangi (Meghe), Wardha.

## Materials and methods

After obtaining ethical approval from the Institutional Ethics Committee (DMIHER(DU)/IEC/2023/1072), Sharad Pawar Dental College and Hospital, DMIHER, a descriptive, cross-sectional study was conducted among healthy relatives of cancer patients who attended their family member admitted in the hospital. The study was conducted for four months, from March 2023 to June 2023. A total of 400 participants, all of them above the age of 18 years, participated in the study.

The questionnaire comprised 23 questions which were modified and finalized according to the responses from the relatives. All the questions were written in the local language (Marathi), which was well understood by the patients, and, if and when needed, were translated for them. All respondents received guarantees of secrecy and anonymity. The participants were given an explanation of the study's goals and methodology in the language they were comfortable speaking. After receiving permission, the relatives were taken to a distinct counseling room aside from the patient in issue, where they were asked to sign a consent form in their original mother tongue before the evaluation could start. After getting a signed agreement, the families were questioned regarding their knowledge, opinions, and attitudes toward various aspects of cancer.

The interview was based on a 23-question questionnaire that was both open-ended and closed-ended (yes/no), which was conducted in their native mother tongue. The questionnaire was formulated in two sections; the first section included open-ended questions regarding the demographic details, which mainly comprised of the patient's in-patient department (IPD) number and the relative's age, gender, marital status, income, education, occupation, number of children and number of dependents. The second section was of both open-ended and closed-ended questions; it comprised 23 questions, out of which 22 questions were mainly of 'yes' and 'no' type that was required to evaluate the knowledge and attitude of the patient's relatives regarding cancer, and only one question was kept open-ended to know the duration from which the patient was suffering from cancer.

Statistical analysis

Statistical analysis was done by SPSS version 23 (IBM Inc., Armonk, New York). Descriptive statistics and frequency distribution was done for determining the mean values and standard deviation. Chi-squared statistics was done to determine the association of demographic variables with the questionnaire. Statistical significance was kept at p<0.05.

## Results

Demographic details of the participants under study

Demographic data about the study's participants were mentioned in Table 1, which shows that maximum participation was from the age group <35 years, then from <50 years, and then lastly from 55-85 years. It was observed that the participation of females was slightly higher than males with not much significance (50.5% females contrary to 49.5% males). In the study, the maximum number of relatives were married (62.3%), that is, 50% of the total participation, and the rest 50% were unmarried (32.8%), divorced (0.5%), or widowed (4.5%) combined. It was found that almost 70% of the families had several children ranging from two to five (<2 was 27.1% and 2-5 was 72.9%), and hence, the total number of dependents on the head of the family was higher. A significant number of participants were university graduates (47.5%), and others had completed secondary school (39.5%), high school (7%), middle school (3.7%), primary school (2%), and the rest were uneducated (0.2%).

**Table 1:** Demographic details of the population under study (n=400)

Variable	Number (%)
Age group	
<35 years	210 (52.5%)
<55 years	135 (33.7%)
55-85 years	54 (13.5%)
Gender	
Male	198 (49.5%)
Female	202 (50.5%)
Marital status	
Married	249 (62.3%)
Unmarried	131 (32.8%)
Divorced	2 (0.5%)
Widowed	18 (4.5%)
Number of children	
<2 children	108 (27%)
2-5 children	288 (73%)
Education	
University graduates	190 (47.5%)
Secondary school	158 (39.5%)
High school	28 (7%)
Middle school	15 (3.7%)
Primary school	8 (2%)
Uneducated	1 (0.2%)

Knowledge about oral cancer

**Figure 1:**
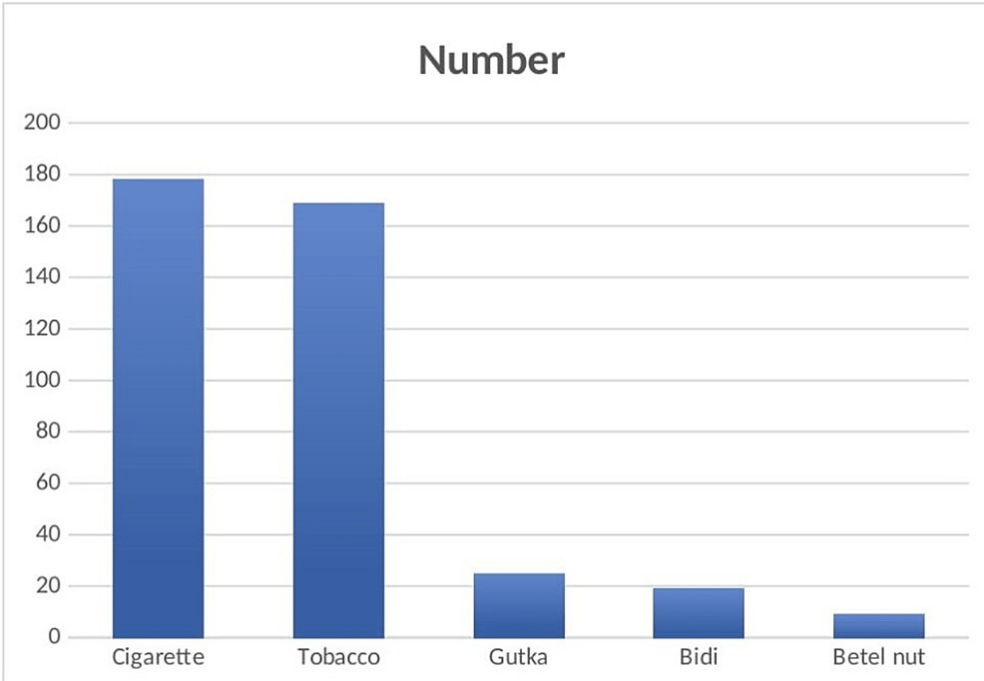
Frequency distribution of the causes of oral cancer

As shown in Table 2, a significant proportion of patients' relatives learned about oral cancer via media such as television, newspapers, radio, and books/magazines, while a smaller number learned about it from Anganwadi workers, healthcare workers, and primary health care centers. Participants in my study believed that smoking cigarettes and chewing tobacco, along with bidi and gutka, were the main causes of oral cancer (Figure 1). Correlating participant demographic data with the questions posed to them is essential since doing so enables us to establish whether or not there is a statistically significant difference between the two (Table 3).

**Table 2:** Frequency distribution of the source of information about oral cancer

Source of information	Number (%)
Television	202 (63.5%)
Newspaper	140 (35.0%)
Radio	15 (3.8%)
Books/magazines	21 (5.3%)
Anganwadi worker	6 (1.5%)
Health care worker	11 (2.8%)
Primary health care center	5 (1.3%)

**Table 3:** Association of demographic variables with the questionnaire

Sr no.	Questions	p-value						
		Age	Gender	Marital status	No. of children	Education	Occupation	Income
1	How did you find out about oral cancer?	0.571	0.077	0.03	0.161	0.01	0.411	0.001
2	What is the main cause of oral cancer?	0.426	0.933	0.024	0.008	0.285	0.352	0
3	Do you think cancer is preventable?	0.079	0.52	0.034	0.227	0.033	0.653	0.002
4	What was the real reason for consulting the doctor?	0.23	0.551	0.69	0.85	0.658	0.171	0.296
5	Do you think it is too late to consult a doctor?	0.362	0.212	0.102	0.922	0.131	0.908	0.007
6	If yes, can the current disease worsen due to delay?	0.895	0.33	0.016	0.926	0.721	0.137	0.004
7	Did you understand the information given by the doctor, nurse, attendant about the disease?	0.003	0.805	0.704	0.174	0.143	0.187	0.093
8	Do you know the treatment methods for oral cancer?	0.064	0.626	0.542	0.198	0.168	0.065	0.006
9	Do you think oral cancer can be cured?	0.05	0.136	0.074	0.26	0.068	0.083	0.049
10	Do you know the side effects of oral cancer after treatment?	0.057	0.734	0.069	0.532	0.029	0.202	0
11	How does it feel to be with a relative who has cancer?	0.005	0.697	0.537	0.3	0.001	0.03	0.001
12	What problems did you face after giving up betel nut/ tobacco/ kharra?	0.075	0.213	0.006	0.138	0.005	0.402	0.001

Perception of relatives about cancer

Almost half of the healthy relatives answered that cancer is preventable (58.0%), whereas others answered 'no' (22.8%) and 'don't know' (24.3%). Most of the participants already knew about the disease their relative was suffering from (60.3%), and 39.8% of them were still not sure. Nearly 46.5% of the patient's family members visited the doctor for the patient's non-healing ulcer; the remaining 11.5% did so for pain, and 21.0% did so for both swelling both inside and outside the mouth. Almost half, or 51.8%, of the participants agreed that they had a close family who had previously battled cancer, while 48.3% disagreed. Out of all participants, 78.5% knew the type of oral cancer their relative had; of these, 57.0% believed it was caused by betel nut or tobacco, 21.5% disagreed, and the remaining 21.4% were unsure. The information provided by the physicians, nurses, and attendants was comprehended by nearly all of the relatives (69.0%), with the remaining 31.0% not following. It was discovered that 51.0% of the participants were not aware that cancer may spread to other bodily areas, 4.0% were aware, and the remaining 6.0% did not believe it could. The information of the participants who were in good health regarding the treatment of patients with oral cancer is shown in Table 4. This knowledge covers whether oral cancer can be treated, treatment options, oral cancer recurrence, and side effects of medication after treatment.

**Table 4:** Assessment of knowledge of participants regarding the treatment of oral cancer

Questions related to treatment	Options	Number (%)
Do you think oral cancer can be cured?	Yes	264 (6.0%)
	No	136 (46.0%)
Do you know the treatment methods of oral cancer?	Yes	171 (42.7%)
	No	229 (57.3%)
Can cancer recur after treatment?	Yes	167 (41.8%)
	No	66 (16.4%)
	Don't know	167 (41.8%)
Do you know the side effects of the medication of oral cancer after treatment?	Yes	149 (3.3%)
	No	50(12.5%)
	Don't know	20(50.3%)

Most of the patients find it difficult to live with the patients suffering from oral cancer (48.7%), others find it sympathetic (38.8%), and a few find it okay (12.5%). The next final set of questions focuses on participants' perceptions, challenges, and decisions related to the intake of cigarettes, tobacco, betel nuts, etc. Among the participants, 30.4% stated that they consumed betel nuts, 8.3% reported consuming cigarettes, and 4.0% reported drinking alcohol, while the remaining 57.3% reported not consuming any of these substances. Nearly all of the relatives who consume items containing cancer-causing agents experience specific issues, such as a burning sensation in the mouth (4.3%), a white patch in the mouth (44.6%), stiffened cheeks (44.1%), and difficulties swallowing (3.0%). And amongst them, 56.0% considered quitting the habit after learning about the difficulties their admitting relatives faced, while 43.8% chose not to. Out of this, 56.0% of the family members, 8.0% sought medical counsel to quit, 45.0% disregarded the advice of a doctor, 51.0% stopped eating, and 3.3% resumed eating after stopping. Last but not least, of the 56% of individuals who stopped eating, some had withdrawal symptoms, such as constipation (32.6%), difficulty focusing at work (33.0%), and trouble sleeping (34.4%).

## Discussion

Knowledge and attitude are the main requirements among the relatives of patients if they are involved in the decision-making process for the treatment of oral cancer patients. In this study, it was found that the relatives of patients who were among the educated group tended to make better decisions which led to a reasonable prognosis of oral cancer. Similar results were found in a study conducted by Kizhakkoottu et al. (2020), which states that the prognosis and treatment protocol of oral cancer patients might be affected by the understanding and knowledge of family members about the disease. Early diagnosis and higher patient recovery rates will result from good family education [4,8]. The incidence of oral cancer among males was found to be higher than among females in a study conducted by Conway et al. and Edward et al. [9,10]. They also stated the relationship of alcohol consumption and smoking to the incidence of oral cancer, in which it was found that the low socio-economic strata tend to consume more alcohol and smoke more as compared to the upper class, which has led to a higher number of oral cancer cases among them. Higher levels of education were found to be associated with a better scientific understanding of cancer and its different components, such as prevention and treatment [11,12].

A majority of the healthy participants were acquainted with oral cancer and knew about it, majorly through television and newspaper. This indicates that mass media plays a crucial role in spreading awareness among the population about oral cancer. Also, the advertisement shown on television and in movie theatres before starting a movie has a great impact. Reddy et al. (2012) and Maweri et al. (2017) also discussed the same in their respective research [7,13]. The majority of research participants think that either cigarettes or tobacco are to blame for cancer, and a few believed that bidi, gutka, or betel nuts were the primary causes of oral cancer, which indicates a lack of understanding of carcinogens as these substances are also cancer-causing substances. But still, some participants believe that this is not the cause, and hence, awareness among the population is fairly low. The result of our research also showed that almost all the participants think that cancer can be prevented but do not know the lifestyle they should have to prevent it. Hence, awareness among them is lacking, and the government should take appropriate measures to spread knowledge regarding the same.

In this study, it was discovered that most relatives found it difficult (48.8%) and empathetic (12.5%) to live with oral cancer patients, which had a huge impact on their personal and professional life. A similar result was also stated by Schaller et al. (2014) in their study, where the relatives found it difficult to manage their job as well as take care of the patients [14]. A study done by Hiremath et al. (2017) also showed that the caregivers of cancer patients show high levels of stress and high burden or moderate to severe burden are common in caregivers of cancer [15,16]. A study was also conducted in Trivandrum, which stated the familial relationship of oral cancer. According to them, even the family members without vices like smoking, drinking, or chewing tobacco were affected [17]. In this study, the familial aggregation was found to be negligible, and hence, the cause of oral cancer was purely due to the consumption of smoke or smokeless tobacco.

In addition, it was found that most of the healthy relatives did know about the type of cancer the patient was suffering from, out of which 50% were informed by the medical staff after admission of the patient to the hospital, and the other 50% knew beforehand. Even though 51.8% of the healthy participants reported having a close relative with cancer and even the majority of the relatives were aware of the disease, 70.3% of them feel that they delayed seeking medical attention for their own family member's oral cancer, and 69.0% of them thought that it led to the worsening of the current disease and this illustrates the pessimistic and belligerent attitudes of the relatives towards cancer. However, even after the delay had occurred, only 29.8% of them believed that they had sought medical attention promptly. This suggests the lack of awareness about the consequences that this deadly disease might cause, which in extreme conditions, leads to death.

Almost half of the patients did not know that cancer may spread to other bodily regions, and the majority of the relatives did not know the treatment options for oral cancer. And if not appropriately informed, lack of knowledge can limit the treatment options that the relatives can choose from for the enhanced quality of life of the patient and to have a good prognosis of the disease at a cost that they can afford as maximum patients in this study belonged to a low socio-economic stratum. All in all, it was found that relatives play a significant role in the treatment of patients suffering from oral cancer and, therefore, should have appropriate knowledge, perception, and a positive attitude.

Limitations of the study

This study had certain limitations; the relationship of the patients to their relatives was not mentioned, which could not give a proper idea about the involvement of the relatives in the decision-making process. The study was done in a limited geographic area that did not relate to a wider population. Also, this survey could not cover all the aspects regarding the knowledge and attitude of the relatives of patients as there is no standardized set of questions for the same. 

## Conclusions

In this study, it was found that relatives had a major role to play when it comes to the decision-making process in the treatment of the patients; hence, it is gathered that certain protocols are needed to spread awareness among a wide population, especially the oral cancer-prone areas about the risk factor of oral cancer, ways to prevent it and cost-effective treatment plans. They should be made fully aware of the cancer-causing agents, such as tobacco, cigarettes, etc., and the hazards caused by them. Certain educational programs or counseling sessions should be accessible to all and be organized at the institutional level, and government schemes should be made available for patients so that treatment is cost-effective for the relatives. Additionally, as the prevalence of oral cancer among youngsters is increasing, specific information on the disease should be included in the curriculum for students. It is of immense importance that people know the early signs of cancer, which enables early diagnosis followed by proper treatment leading to proper quality of life.

## Appendices

The questions asked of participants are shown in Table 5.

**Table 5:** Questionnaire

Sr no.	Question	Response
1	Have you heard about oral cancer?	Yes/No
2	How did you find out about oral cancer?	Television/Newspaper/Radio/Textbooks/Anganwadi Worker/Health Care Worker/Primary health care centre
3	What is the main cause of oral cancer?	Tobacco/Cigarette/Bidi/Betel nut/Gutka
4	Do you think cancer is preventable?	Yes/No/Don’t know
5	Do you know the disease of your relative?	Yes/No
6	How long has your relative been suffering?	
7	What was the real reason for consulting the doctor?	Non-healing ulcer/Pain/Swelling inside the mouth/Swelling outside the mouth
8	Have any of your close relatives ever had cancer?	Yes/No
9	Do you think it is too late to consult a doctor?	Yes/No
10	If yes, can the current disease worsen due to delay?	Yes/No
11	Do you know what type of oral cancer your relative has?	If yes, then due to betel nut or tobacco/If yes, then not due to betel nut or tobacco/No
12	Did you understand the information given by the doctor, nurse, attendant about the disease?	Yes/No
13	Does oral cancer spread to other parts of the body?	Yes/No/Don’t know
14	Do you know the treatment methods of oral cancer?	Yes/No
15	Do you think oral cancer can be cured?	Yes/No
16	Can cancer recur after treatment?	Yes/No/Don’t know
17	Do you know the side effects of the medication of oral cancer after treatment?	Yes/No/Don’t know
18	How does it feel to be with a relative who has cancer?	Ok/Difficult/Sympathetic
19	Do you consume betel nuts/tobacco and alcohol?	Yes, then only Betel nut/Yes, then only Tobacco/Yes, then only alcohol/No
20	What problems did you face after eating betel nut/kharra?	Burning sensation in mouth/White patch in mouth/Cheeks hardened/Change in voice/Difficulty in swallowing
21	Have you thought of stopping the habit after seeing the trouble your relatives are going through?	Yes/No
22	If yes, then what did you do?	Consulted a doctor to quit/Did not take doctor's advice to quit/Stopped eating/Started eating again after quitting
23	What problems did you face after giving up betel nut/tobacco/kharra?	Not able to concentrate at work/Not able to sleep/constipation
